# Supplementary material for: Scalable Apparatus to Measure Posture and Locomotion (SAMPL): a high-throughput solution to study unconstrained vertical behavior in small animals
Source: bioRxiv. 2023 Mar 27:2023.01.07.523102. Originally published 2023 Jan 7. Preprint. [Version 2] doi: 10.1101/2023.01.07.523102 (PMC9881893; doi:10.1101/2023.01.07.523102)
Supplement: Supplement 4 [file NIHPP2023.01.07.523102v2-supplement-4.pdf]

bioRxiv preprint doi: https://doi.org/10.1101/2023.01.07.523102; this version posted March 27, 2023. The copyright holder for this preprint (which was not certified by peer review) is the author/funder, who has granted bioRxiv a license to display the preprint in perpetuity. It is made available under aCC-BY 4.0 International license.

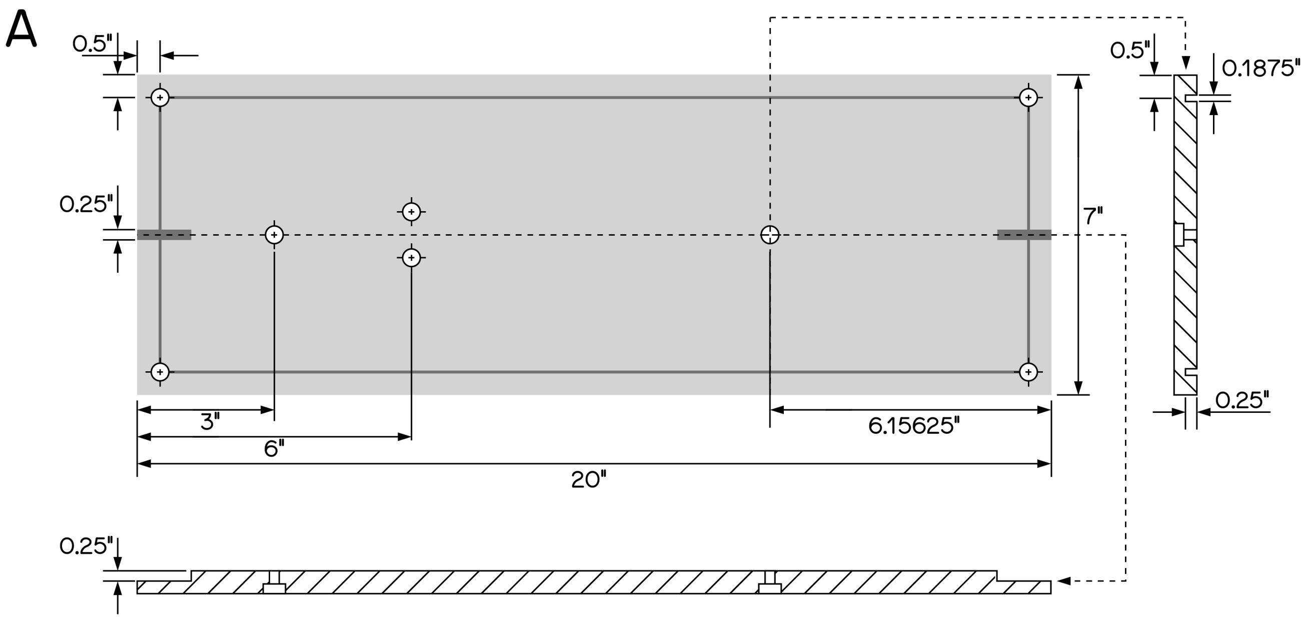


**Figure S1: Custom breadboard for SAMPL base**

(A) Custom aluminum breadboard, not anodized, 0.5" thick. All holes (8 total) counterbored for 1/4"-20 cap screw. Grooves to be cut on the side of the breadboard OPPOSITE to the counterbore.
