# Supplementary material for: Scalable Apparatus to Measure Posture and Locomotion (SAMPL): a high-throughput solution to study unconstrained vertical behavior in small animals
Source: bioRxiv. 2023 Jan 7:2023.01.07.523102. Preprint. [Version 1] doi: 10.1101/2023.01.07.523102 (PMC9881893; doi:10.1101/2023.01.07.523102)
Supplement: Supplementary file 1 [file NIHPP2023.01.07.523102v1-supplement-4.pdf]

bioRxiv preprint doi: https://doi.org/10.1101/2023.01.07.523102; this version posted January 7, 2023. The copyright holder for this preprint (which was not certified by peer review) is the author/funder, who has granted bioRxiv a license to display the preprint in perpetuity. It is made available under aCC-BY 4.0 International license.

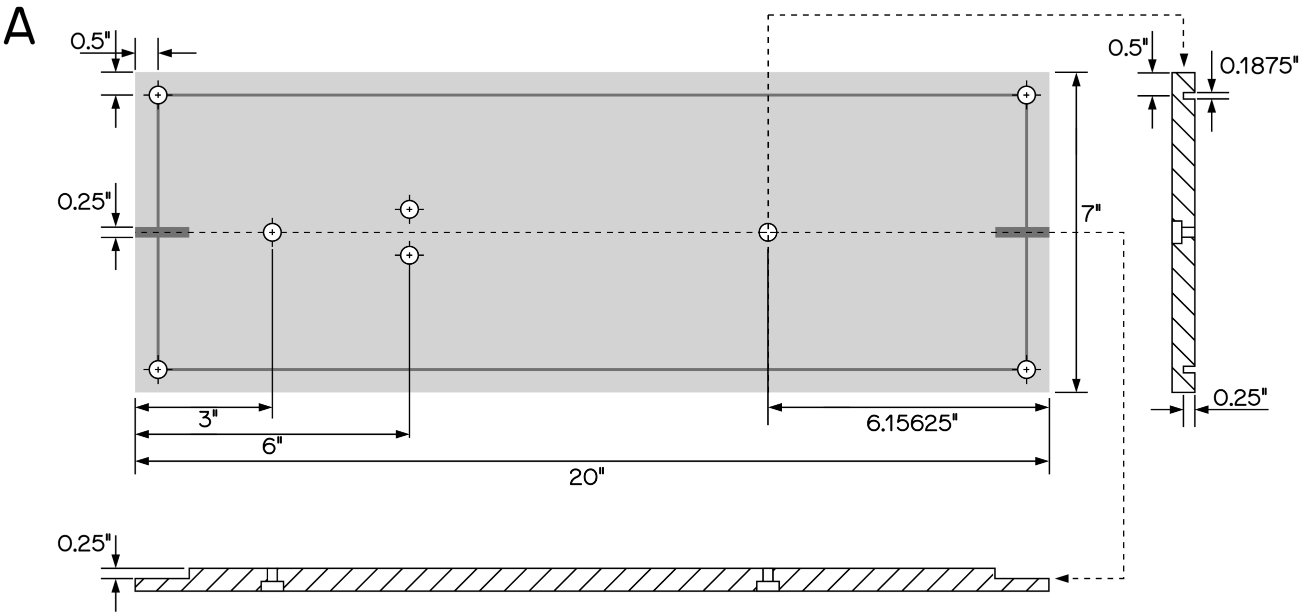

Figure S1: Custom breadboard for SAMPL base
(A) Custom aluminum breadboard, not anodized, 0.5" thick. All holes (8 total) counterbored for 1/4"-20 cap screw. Grooves to be cut on the side of the breadboard OPPOSITE to the counterbore.
